# Well-Being During Recession in the UK

**DOI:** 10.1007/s11482-016-9465-8

**Published:** 2016-04-29

**Authors:** David Bayliss, Wendy Olsen, Pierre Walthery

**Affiliations:** 1 Cathie Marsh Institute for Social Research, University of Manchester, Manchester, UK; 2 University of Oxford, Oxford, UK

**Keywords:** Well-being, Life satisfaction, Positive psychological health, Recession, Latent curve model

## Abstract

This article explores the impact of the recent recession on the well-being of the UK working age population by comparing two measures of well-being. One is a measure of evaluative subjective well-being, a measure which previous research has shown to be stable in the UK throughout the economic crisis. The second is a different but complementary measure of positive psychological health. By comparing the trajectories of these two measures using the same sample and modelling techniques the analysis examines how different measures may lead to different interpretations. Six waves of longitudinal data from Understanding Society and the British Household Panel Survey (BHPS) are used. Latent curve models are used to analyse change over time. The results corroborate previous research showing that people’s evaluative subjective well-being remained relatively stable, on average, throughout the economic crisis. In contrast, the positive psychological health measure was found to decline significantly during the recession period. The paper highlights that what we measure matters. Using single measures as summaries of well-being masks the complexity of the term, and given their appeal in the social policy arena, single measures of well-being can be seen as problematic in some scenarios.

**Electronic supplementary material:**

The online version of this article (doi:10.1007/s11482-016-9465-8) contains supplementary material, which is available to authorized users.

## Introduction

An understanding of the impact of recession on people’s well-being is important to inform social and economic policy. A small body of work directly addresses the question of the impact of the 2007/8 economic crisis on well-being in the UK. This work based on a variety of sources finds that aggregate well-being remained stable. Well-being is a broad and multidimensional concept which can be operationalised in many different ways. Since different approaches to well-being may result in varied interpretations, investigating the implication of conceptual and operational choices is important. The approaches which have generated the findings that well-being remained stable during recession are based on singular ‘evaluative’ measures of subjective well-being (SWB), measures common within economics and psychology (Dolan et al. 2008). This paper aims to test whether this conceptualisation and operationalisation of well-being leads to the description of stable well-being. We do this by comparing *life satisfaction* (a SWB measure) with an alternative but complementary measure of *positive psychological health*, hypothesising that the latter will have responded to the economic crisis.

### Well-Being and Recession

An empirical connection between macroeconomic circumstances and well-being is regularly made in the economic literature. Di Tella et al. (2001) use repeated cross-sectional data from 12 European countries to show that, at an aggregate level, people are happier when inflation and unemployment are low, and that of these measures unemployment has the largest effect. Blanchflower et al. (2014) also finds that interest rates are negatively associated with well-being, and in poorer countries GDP per capita is found to be positively associated (Blanchflower 2007). An economic crisis is by definition extreme change in macroeconomic conditions and so well-being ramifications may be expected on these grounds. Indeed, Clark (2011a) documents a decline in well-being associated with the early 1990s recession in Britain. There are many mechanisms through which recession could impact on well-being by altering people’s living conditions. These range from financial pressures and losing employment (Mandemakers and Monden 2013; Gush et al. 2015) to reductions in socially provided services (Meegan et al. 2014). Well-being may also be affected by subjective expectations of what the recession might mean for individuals, connected more to the events of the economic crisis and our cognitive response to it than changes in living conditions, as Deaton (2012: 22) found using daily cross-sectional data for the United States from January 2008 to December 2010: “perhaps the most surprising finding is how closely well-being tracked the stock market...My guess is that the stock market became the most watched indicator” and that people’s fears were “heavily reinforced by media coverage, sending highly correlated interpretations of events to a large segment of the population.” The result was that population average well-being had dropped and recovered by 2010.

**Fig. 1:**
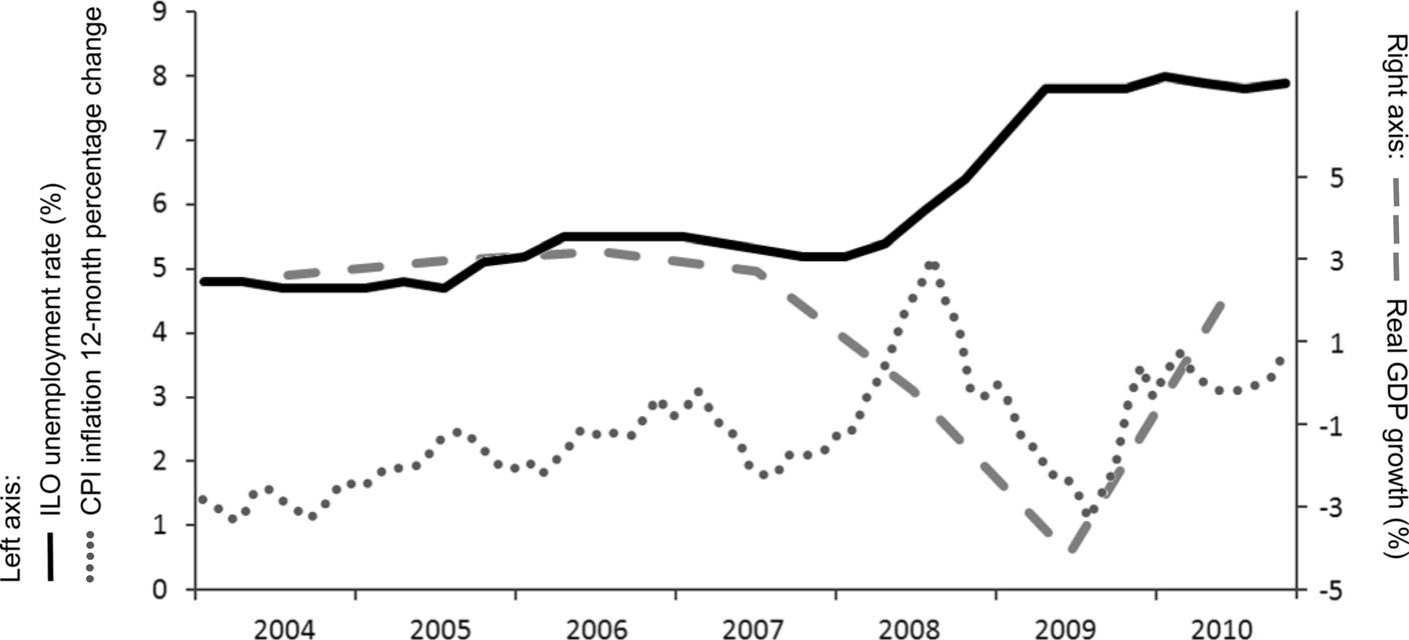
Time series of unemployment rate (NOMIS 2011), CPI inflation (ONS 2011) and real GPD growth (World Bank 2015) over the study period

Both changes in living conditions and the more immediate impact of subjective response to the economic crisis are plausible mechanisms. These mechanisms will exert themselves at different times and in different groups of society during the recession period. And while UK data does not allow us to replicate the detailed analysis of Deaton (2012) and therefore attempt to disentangle the mechanisms, it is worth briefly looking at how these may have played out in the UK. Awareness of worsening economic conditions in the general public may have started as early as 2007 with a series of bankruptcies in the US, followed by the high profile collapse of Northern Rock in the UK (September 2007). Throughout 2008 further high profile events occurred as the UK government bailed-out several banks and similar actions took place in the United States (as detailed by Graham et al. 2010). October 2008 saw stock markets plummet after having declined slowly since late 2007, staying low until mid-2009, but largely recovering by early 2010 (Google Finance 2015). This is reflected in real GDP growth, presented in Fig. 1 with key time-series trends which highlight widespread coinciding change. Inflation started rising in early 2008, peaking initially in September 2008 before a dramatic drop and subsequent rise over the next 3 years (ONS 2011). The ONS (2009) places the onset of recession in the UK during the second quarter of 2008 (based on two consecutive quarters of negative growth), coinciding with increasing unemployment rates which did not start to level off until the beginning of 2010 (NOMIS 2011). Announcement of official recession necessarily lagged, occurring in November 2008 for the Eurozone, followed by the US in December and UK in January 2009 (BBC 2009). Changes in public service provision and welfare conditions as a result of austerity measures were of relatively small scale in the initial years after recession. However, even before new welfare regimes were implemented (often several years after the new government in 2010 and therefore outside the study period), the punitive rhetoric against ‘welfare dependency’ and ‘scroungers’ to garner public support for austerity could also have acted to reduce the social and financial security in some sections of society (Hennessy 2012; Mooney 2011).

All these recession-related events and more could potentially influence well-being. Subjective responses to these negative events may be expected to impact well-being in the early recession period, whereas changes in lived conditions may take longer to materialise. The impact of the 2007/8 economic crisis on well-being in the UK has been directly addressed in just a few studies. Crabtree’s (2010) report based on a Gallup poll highlights the stability of British people’s well-being, with the score on a scale from 0 to 10 hovering around seven annually between 2005 and 2010: “Britons’ wellbeing has neither significantly improved nor deteriorated in recent years...despite 5 years of economic turmoil that included the country’s longest recession on record.” It is a similar message to that which has emerged in reports from the UK Office for National Statistics (Self et al. 2012; ONS 2013) reporting of ‘personal well-being’. Data from the World Database of Happiness (from 2002 to 2011) finds that “life satisfaction remained broadly stable throughout the last decade” (Self et al. 2012: 11), its mean again hovering around seven on a 0–10 scale. In *Personal Well-being in the UK* (ONS 2013: 10), there is another description of a “picture of stability in life satisfaction in the UK,” supported by data from the European Quality of Life Survey in which this measure of personal well-being is unchanged in the UK from 2007 to 2011, in contrast to other European countries. Comparing these results to Deaton’s (2012) study (leaving aside potential differences arising from study designs), the lack of change may suggest the UK differed to the United States.

One explanation for the stable average well-being of the UK population is a focus on aggregate analysis. The negative impacts of recession may be focussed on a small subsection of the population and balanced out by improvements in well-being in other sections of society due to lifestyle changes and reductions in external sources of death (such as traffic fatalities). This argument has been made from a health perspective by Burgard et al. (2013) and Ruhm (2013) who suggest that population level changes in health due to economic crises are likely to be positive in the form of an overall reduction in mortality. This conclusion is based on those not directly affected by recession experiencing small health benefits and outnumbering the smaller number who are directly affected but experience larger health costs. Aggregate analysis will always mask heterogeneity across the population. However, given the larger array of ways recession could impact on well-being compared to mortality, including through subjective responses, this explanation of aggregate stability is less persuasive.

Another explanation for stable well-being is the operationalisation and conceptualisation of well-being. The studies discussed above are based on analysis of subjective well-being (SWB) measured predominantly using evaluative measures of satisfaction (discussed further below). Looking to less direct studies, much of the research exploring the impact of recession in the UK focuses on health outcomes rather than well-being more broadly. Depending on one’s conception of well-being, health outcomes are either a strong predictor or a central constituent of well-being at the individual level. Katikireddi et al. (2012) plot the prevalence of poor mental health from 1991 to 2010 using cross-sectional data from the Health Survey of England, and find that compared to 2008, the prevalence of poor mental health was higher in 2009 and to a lesser extent 2010, for men only. Though the authors point to question ordering effects as potentially biasing the 2009 findings, Spence et al.’s (2014) findings that prescriptions of medication related to depression and anxiety increased during the recession period support their conclusion. Average self-reported health in the UK has also declined during the recession period according to Astell-Burt and Feng (2013). The difference between studies measuring SWB and those looking at more specific dimensions of well-being may provide insight into why well-being has reportedly been stable in the UK.

### Measuring Well-Being

The concept of well-being is broad and multidimensional and as a result has been conceptualised and operationalised in a variety of ways within empirical research across and within disciplines (for the taxonomy of well-being and related concepts, see Anand et al. 2009; Burchardt and Vizard 2011; Gasper 2010; McGillivray 2007). Diener et al. (1999: 277) describes SWB as “a general area of scientific interest rather than single specific construct,” and Jivraj et al. (2014) suggest that evaluative SWB is one of three approaches to studying SWB (with affective and eudaimonic making up the others). Advocates of SWB measures consider these measures as the outcome of a broad range of factors which may be considered *determinants* of well-being. This contrasts with advocates of multidimensional approaches to studying well-being, who typically see the broad range of factors as *constituents* of well-being (Austin 2015).

Crabtree’s (2010) analysis of well-being through recession uses a question which asks respondents how they feel about their life compared to an imagined ‘best possible life for you’ (the Cantril Self-Anchoring Striving Scale). A similar but more direct question is the more typical ‘life satisfaction’ question used as one of four ‘personal well-being’ subjective measures adopted by the ONS (Self et al. 2012; ONS 2013): “Overall, how satisfied are you with your life nowadays?” These questions are subjective measures of subjective concepts. Not only is the measurement process subjective, but the content and scope of factors which constitute satisfaction or the ‘best possible life’ may well be different for each subject and change over time. Plagnol and Scott (2011) argue such instability exists, giving evidence of change over the life course and differences between genders in the conceptualisation of well-being. It is possible that the nature of evaluative SWB questions is less well suited to recording the complexity of changes in lived conditions associated with the recession. One reason may be adaptation.

Adaptation, in which our subjective assessments of our lives adapt according to external changes in conditions, is well noted (Nussbaum 2001; Diener et al. 2006). But we refrain from presenting adaptation as a blanket explanation as to why evaluative SWB seems not to have responded to the recession because empirical work demonstrates that adaptation occurs to varying degrees in response to different events (Luhmann et al. 2012; Clark 2014). It has been shown for example, that evaluative SWB does not adapt back to a baseline level after being made unemployed (Burchell 2011; Clark and Georgellis 2013). The proportion of unemployed people even during this severe recession is relatively small, however job insecurity increased sharply during the recession (Gallie et al. 2014), which is associated with lower levels of well-being but no process of adaptation (Burchell 2011). In contrast adaptation to change in income in some cases is complete, returning to a baseline (Clark 2014), yet adaptation to poverty (defined by income) is not (evidence from Germany, Clark et al. 2016). Adaptation then may be a partial explanation for stable SWB during the recession.

Evaluative SWB measures co-vary with many important life events, demonstrating their validity and scientific worth for well-being research and social policy (e.g. Luhmann et al. 2012; Yap et al. 2014). The purpose of this paper is to highlight a prominent case in which the overreliance on measures of evaluative SWB is problematic. In doing so we advocate a multidimensional conception of well-being and take the view of Sen (1987) who suggests that SWB measures such as happiness and life satisfaction have value in the study of well-being only if it is recognised that they are only one constituent part, since they are not the only items of value. Understanding the relationship between recessions and well-being is necessary for governance. An understanding of how the population is affected enables informed social policy decisions. Furthermore, understanding the implications of macroeconomic change, which is manipulated through policy levers, similarly enables more informed decision making. That decision making may be impaired by reliance on evaluative SWB measures, which to date have produced positive summary statements about well-being holding steady in the UK.

In this paper we ask whether the recession had a negative impact on the well-being of the UK working age population between 2004 and 2010, and if the conceptualisation and operationalisation of well-being as life satisfaction is responsible for the narrative of stable well-being. To understand the well-being implications of the recession we suggest it is most informative not to restrict analysis to evaluative SWB measures, and put forward a related but distinct measure of *positive psychological health* with which to explore the well-being impact of recession. This measure (discussed below) does respond to the recession period and so helps to uncover why previous analyses may not have.

## Methodology

### Sample

Central to exploring how individual well-being has changed over time is the use of secondary longitudinal data that covered the period of the recession. This was provided by Understanding Society and the British Household Panel Survey (BHPS). These large scale surveys use an annual panel design and are therefore especially well suited to analysing how people experience and respond to change in their socioeconomic environment (Taylor et al. 2010). The BHPS ended in 2008 being replaced by Understanding Society which is a different but complementary survey which incorporates the BHPS sample within it. This has to some extent enabled individual level analysis to continue, though much potentially useful data is not comparable across the two surveys. The BHPS sub-sample remains independent of the main Understanding Society sample, therefore it is the BHPS survey design which is key to analysing the combined dataset, a detailed account of which can be found in Taylor et al. (2010).

Analyses are run on six survey waves from 2004 to 2010 (2009 omitted as the BHPS sub-sample are not included in this wave of Understanding Society) with three waves in the pre-recession period and three waves in the recessionary period. The data is primarily conducted through face-to-face surveys, though includes a self-completion questionnaire which captures one of the key variables used in this study. The population of interest for the study is people of working age in the UK, excluding fulltime students, people who have retired and those in government training schemes. Working age is defined as 16–59 years for women and 16–64 years for men. The analysis tracks the sample from the 2004 wave over the subsequent five waves, allowing respondents to drop out and return or exit, creating an unbalanced dataset. The sample size is 10,260 respondents who provide 46,751 observations over the study period (a description of sample characteristics can be found in the Online Resource 1).

### Measures

The analysis compares two outcome variables. One is *life satisfaction*, an evaluative measure of subjective well-being very similar to that used in the aforementioned research which suggests well-being has remained stable during the recession period (ONS 2013; Self et al. 2012). The other is a measure of *positive psychological health* which is derived from the 12-item General Health Questionnaire (GHQ-12), discussed below. Though evidence of stable *life satisfaction* has been replicated, comparing these two measures is necessary so that any differences observed can be attributed to the measures themselves and not differences in sample or study design. *Life satisfaction* is measured using the question “how dissatisfied or satisfied are you with your life overall”, and respondents answer on a scale from 1 (not at all satisfied) to 7 (completely satisfied). Six respondents had item-missing data on this variable (and valid data for the psychological health variable) and were dropped from this analysis reducing N to 10,254. A summary of the variable is presented in Table 1.

**Table 1:** Description of outcome variables: *life satisfaction* and *positive psychological health*

	Life Satisfaction	Positive Psychological Health
At wave 1 in 2004		
Mean	5.13	0.03
Standard deviation	1.17	0.61
Minimum	1	−2.54
Maximum	7	1.94

Population aged 16–59/64 (F/M). BHPS UK sample, wave 2004. Weighted estimates, standard errors adjusted for within-person dependency by multilevel structure. Life satisfaction lay on its natural scale 1–7. Positive Psychological Health lay on an approximately normal distribution curve with most cases lying from −2 to +2, and was constructed using the six ordinally measured GHQ items

The measure of *positive psychological health* is based on six items from the GHQ-12 contained in the self-completion section of the main questionnaire. Huppert and Whittington (2003) argue that the measurement of positive psychological symptoms in the GHQ is not simply the inverse of the negative psychological symptoms. The authors put forward a strong case that the two are not only theoretically distinct (though clearly related), but also empirically different with regard to their distribution in the population and to related predictors. Several studies have also suggested that the factor structure of the GHQ supports a multidimensional solution (Graetz 1991; Rajabi and Sheykhshabani 2009; Vanhoutte 2014). Using the GHQ-12 as a single measure of psychological health is therefore rejected in favour of the more theoretically coherent distinction between positive and negative traits.

A *positive psychological health* variable was created using the six items of the GHQ-12 which measure positive traits.^1^ The ordinal items were analysed using categorical confirmatory factor analysis (CFA) to statistically test the theorised positive construct. This was preferred over an aggregate classical scale as the approach does not assume error-free measurement of the latent factor (*positive psychological health*) or interchangeability between items. The factor structure was estimated for the six waves simultaneously, with the resulting model exhibiting good fit statistics (RMSEA = 0.014, CFI = 0.98, TLI = 0.98) providing confidence in the measurement of the underlying variable *positive psychological health*. A summary of the resulting variable, which has a mean of near zero by design, is presented in Table 1. The analysis uses a two-stage approach in which the factor scores that emerge from the CFA are treated as observed variables in the latent curve model (described below). This option was preferred over simultaneously modelling the CFA and the latent curve model in one stage, which would require many parameter estimates due to combining multiple categorical observed items with six linked waves of data.

The *positive psychological health* variable has several features that make it a good candidate for comparing against *life satisfaction*. The two variables have similarities that make the comparison meaningful. They both focus on the cognitive state of respondents and are subjective in the sense they are self-reported measures. Clark and Oswald (2006: 6) use *life satisfaction* and the full GHQ-12 measure describing them both as complementary subjective well-being measures. While within the SWB framework set out by Diener et al. (1999) in *Subjective Well-Being*: *Three Decades of Progress*, *life satisfaction* and *positive psychological health* could be described as a cognitive evaluation and positive affect respectively. In contrast, the measures also differ in important ways that make the comparison instructive. Unlike *life satisfaction* which is a subjective assessment where the content and scope of factors which constitute satisfaction may well be different for each person and change over time, the *positive psychological health* measure is a subjective assessment of six questions which are constant between people and over time. It is therefore likely to be less prone to adaptation to recession period mechanisms. In a multidimensional well-being framework the two variables are considered different well-being dimensions, with *life satisfaction* a measure of SWB and *positive psychological health* a sub-dimension of health.

### Covariates

The longitudinal method which follows a cohort across the study period is good for studying change, but changes in the cohort over time due to non-response, attrition and changes in sample characteristics such as ageing may introduce bias. Covariates are added to the model to act as control variables, providing greater confidence that the trajectory of change is reflective of changes in the population and not just the sample. Covariates were selected based upon relationships with well-being established in the literature. These include sex, age band, labour market status, marital status, having children living at home, educational attainment, tenure, disability and household income (details of these variables can be found in the Online Resource 1). Labour market status and household income may also be mechanical in the relationship between recession and well-being. The recession increased unemployment and to a lesser extent economic inactivity in the UK. Both of which are associated with poorer well-being than being employed (Pierewan and Tampubolon 2015; Clark and Georgellis 2013). Recession also affects income directly, through for example, job loss and underemployment, and income is also associated with well-being outcomes (Graham 2011; Clark 2011b).

Many of the variables are time-varying, but have been reworked into time-constant versions for the purpose of this analysis to reduce the model and theoretical complexity. The relationships explored with time-constant covariates represent no more than associations between variables, since no changes are observed with which causal mechanisms can be tested.

### Analytical Strategy

We start with descriptive wave-by-wave unconditional mean estimates of the two measures for comparison, to explore the pattern of change over time and inform the main model. Dependence between the repeated measures is controlled for (through nesting observations within people in a multilevel model) in what is otherwise a repeated cross-sectional approach. However, trends in repeated cross-section may not be typical summaries of trends at the individual level and so are not used for the main analysis. The longitudinal data is used to produce individual trajectories of each outcome variable using latent curve models (LCM) for the main analysis, so the aggregate trajectory is derived from individual trajectories, not simply by connecting cross-sectional averages. This technique plots the underlying latent trajectory, minimising the impact of measurement error by separating residual error from true change, and so is advantageous over techniques which assess change by comparing wave-by-wave change.

Here we implement a piecewise trajectory, a variation of the linear model in which the trajectory of the outcome variable is estimated in a series of ‘pieces’. The shape of the tested trajectory is informed by the theoretical model which suggests the recession period should be associated with a decrease in well-being, and by the wave-by-wave descriptive analysis which will help to identify the pattern of change (e.g. step-change versus steady decrease). The model estimates are robust to non-normality in the distribution of the dependent variable.

## Results

### Descriptive Analysis

**Fig. 2:**
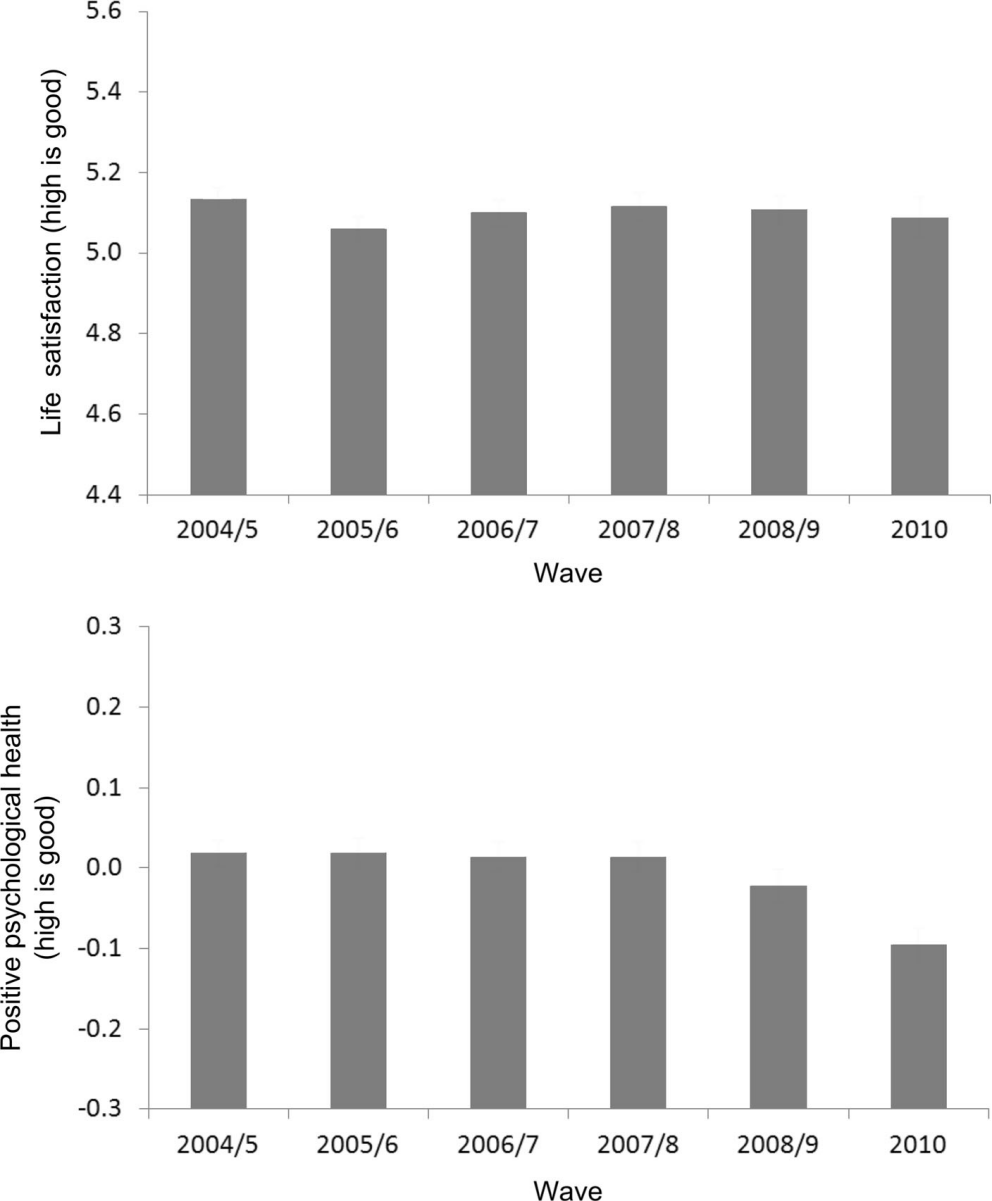
Wave-by-wave estimates of means *life satisfaction* (top panel) and *positive psychological health* (bottom panel), from unconditional multilevel regression

To provide a preliminary look at change over the study period, unconditional wave-by-wave estimates of mean *life satisfaction* and then mean *positive psychological health* were produced. Multilevel regression models in which responses were nested within individuals were used to account for the statistical dependency of repeated observations. The results are presented in Fig. 2, in which the y-axis of each graph is approximately one standard deviation of the respective scale. For *life satisfaction* no clear trend over time is observable in the bivariate values, in line with the ‘stable well-being’ studies discussed earlier. For *positive psychological health* there is preliminary support for the hypothesis that this variable would be relatively stable in the pre-recession period compared to the recession period, in which a steady decline is evident from 0.01 (S.E. = 0.01) in 2007/8 to −0.10 (S.E. = 0.01) in 2010. A steady decline, as opposed to a step-change, suggests that *positive psychological health* is responding to changes in lived circumstances, not (only) subjective reaction to the economic crisis. For example, unemployment (one easily visible objective consequence of recession) increased through 2009 and peaked in 2010. Unemployment is only one recession based mechanism that could impact *positive psychological health*, but it may well be indicative of related changes in people’s conditions and circumstances.

### Main Analysis

The main analysis compares the trajectories of mean *life satisfaction* and *positive psychological health* across the pre-recession and recession periods. Based on the descriptive results above, the piecewise latent curve model defines two slopes (‘pieces’ of the overall trajectory), one representing the pre-recession period (from 2004 to 2006) and the second the recession period (2007–2010). The key parameters in this analysis are the mean latent slopes, which indicate the presence or absence of significant change in well-being over time.

Table 2 presents results from the two piecewise latent curve models. For ease of interpretation, we take advantage of the mean intercept and slope estimates derived from the model provided by the ‘tech 4’ output in Mplus. These mean values are averages across the population and therefore more directly relevant to our hypothesis. They are presented in place of the conditional intercept and slope parameters which are interpreted conditionally upon the average of the continuous covariates and upon the reference categories of the categorical covariates (which are presented alongside the covariates in Table 3).

**Table 2:** Piecewise latent curve models for *life satisfaction* (Model 1) and *positive psychological health* (Model 2), selected output

	Model 1. Life satisfaction^a^	Model 2. Positive psychological health^b^
	Estimate (S.E.)	Estimate (S.E.)
Mean intercept^c^	5.107 (0.02)	0.030 (0.01)
Mean slope (slope 1)^c^	−0.002 (0.01)	−0.002 (0.00)
Mean slope (slope 2)^c^	−0.002 (0.01)	−0.038** (0.00)
Residual variances		
Intercept	0.759 (0.03)	0.187 (0.01)
Slope 1 (pre-recession)	0.038 (0.01)	0.018 (0.00)
Slope 2 (recession)	0.033 (0.01)	0.022 (0.00)
Model fit		
RMSEA	0.008	0.011
CFI	0.995	0.987
TLI	0.990	0.977

Population aged 16–59/64 (F/M). BHPS UK sample wave 2004 to waves 2008 and Understanding Society BHPS cohort wave 2010. Weighted estimates

*p < 0.05; **p < 0.0005

^a^ *N* = 10,254

^b^ *N* = 10,260

^c^These are model estimated means calculated in Mplus based upon model coefficients (obtained using the ‘tech 4’ option in Mplus, see Muthén and Muthén 2012). For example, *model estimated mean of slope* 1 = *intercept of slope* 1 + β_1_ * *mean of* X_1_ + β_2_ * *mean of* X_2_ … β_*k*_ * *mean of* X_*k*_, where k is the number of covariates. It is an aggregate value which accounts for covariates, sample design and weights. Presenting the model estimated means does not alter the empirical model; it is to aid interpretation only. Note: the conditional intercept and slopes and the covariate coefficients are presented separately

**Table 3:** Covariate coefficients for Model 2, piecewise latent curve model for *positive psychological health*

	Intercept	Pre-recession slope	Recession slope
	Estimate (S.E.)	Estimate (S.E.)	Estimate (S.E.)
Constant	0.276 (0.05)	0.005 (0.02)	−0.054*(0.02)
Sex:			
Men^a^			
Women	−0.063** (0.02)	−0.005 (0.01)	−0.004 (0.01)
Age band:			
16–24^a^			
25–34	−0.045 (0.04)	−0.006 (0.02)	0.015 (0.02)
35–49	−0.185** (0.04)	−0.006 (0.02)	0.010 (0.02)
50–59/64	−0.211** (0.04)	−0.007 (0.02)	0.020 (0.02)
Labour market and employment status:			
Employed^a^			
Unemployed	0.001 (0.10)	0.032 (0.05)	−0.013 (0.05)
Economically inactive	−0.231** (0.04)	0.023 (0.02)	−0.009 (0.02)
In and out of employment	−0.081** (0.02)	0.008 (0.01)	−0.004 (0.01)
Between unemployed and inactive	−0.183** (0.05)	−0.018 (0.02)	−0.006 (0.02)
Children in household:			
No children^a^			
Children	−0.005 (0.02)	−0.007 (0.01)	0.011 (0.01)
Marital status:			
Couple^a^			
Single	0.027 (0.03)	0.011 (0.01)	−0.004 (0.01)
Ex-couple	−0.072 (0.04)	0.038*(0.01)	−0.023 (0.02)
Widow/widower	−0.114 (0.08)	0.024 (0.04)	0.018 (0.06)
Educational attainment:			
High^a^			
Intermediate	−0.015 (0.02)	−0.008 (0.01)	−0.001 (0.01)
No qualifications	−0.006 (0.03)	−0.015 (0.01)	0.026 (0.02)
Tenure:			
Owned: outright^a^			
Owned: mortgage	−0.020 (0.02)	0.004 (0.01)	0.004 (0.01)
Rent: social	−0.021 (0.03)	−0.011 (0.01)	0.015 (0.02)
Rent: private/other	−0.065 (0.04)	0.016 (0.01)	−0.019 (0.02)
Disability:			
Not disabled^a^			
Considers self disabled	−0.247** (0.04)	−0.012 (0.02)	0.007 (0.02)
Household income (centred) (log)	0.036* (0.02)	0.007 (0.01)	0.007 (0.01)

Population aged 16–59/64 (F/M). BHPS UK sample, wave 2004 to waves 2008 and Understanding Society BHPS cohort wave 2010. Weighted estimates. Note: The covariate table for Model 1 is presented in Online Resource 2 as it is not central to the research question

*p < 0.05; **p < 0.0005; *N* = 10,260

^a^reference category

**Fig. 3:**
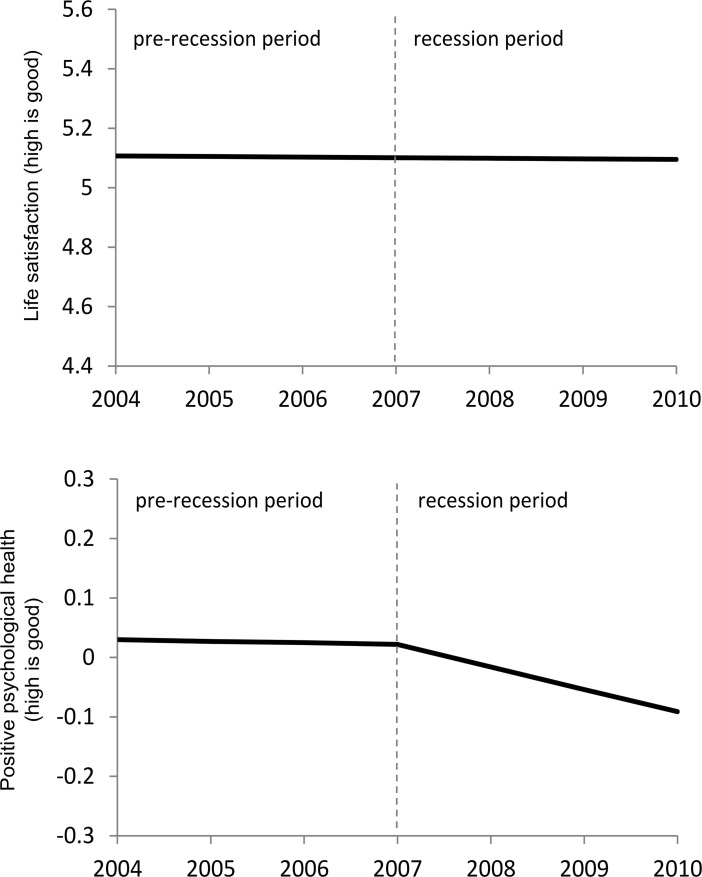
Estimated mean latent trajectories for *life satisfaction* from Model 1 (*top panel*) and *positive psychological health* from Model 2 (*bottom panel*)

Starting with the model for *life satisfaction* (Model 1, Table 2), no evidence is found of significant change over time in either the pre-recession period (mean slope of −0.002, S.E. = 0.006) or during the recession period (mean slope of −0.002, S.E. = 0.009). This pattern of stability supports both the previous research and our hypothesis. The model for *positive psychological health* (Model 2, Table 2) also shows no significant change in the pre-recession mean slope (−0.002, S.E. = 0.003), though in contrast to Model 1, there is a significant decline in *positive psychological health* in the recession period (−0.038, S.E. = 0.004). This further supports the hypothesis that change in this variable would be associated with the recession period. The model estimated mean trajectories for Model 1 and 2 are plotted in Fig. 3 (based on the means in Table 2), with the y-axis for each graph spanning approximately one standard deviation of the respective scales. Overall the goodness of fit measures show strong support for both models fitting the data (Model 1 for *life satisfaction*: RMSEA = 0.01, CFI = 0.99, TLI = 0.99; Model 2 for *positive psychological health*: RMSEA = 0.01, CFI = 0.99, TLI = 0.98).

### Individual Variation

We now focus in on the model for *positive psychological health* (Model 2) and explore the individual trajectories of change over the study period, thus providing a richer description of change.

Table 3 presents the covariate coefficients from Model 2. The intercept regression is similar to a grand means model, and the factors affecting the slope can be seen as those which significantly affect trajectories. The first column of Table 3 presents the covariate coefficients relating to the intercept. The results show that lower levels of *positive psychological health* are associated with women, people in older age bands (35–49 and 50 plus), those that considered themselves disabled, and people with lower average household income. The consistently economically inactive, people who are in-and-out of employment, and people who move between unemployment and economic inactivity during the study period also have lower levels of *positive psychological health* than those who were employed across the six waves. Respondents who were consistently unemployed however, did not have significantly different results to the consistently employed (though this may be due to the small sample in this category). Around half the variance in the intercept factor is explained by the initial level and set of covariates (intercept R-square = 0.55, not presented), leaving significant unexplained between-person variation (presented in Table 2, residual variance = 0.187, S.E. = 0.010). There are potentially additional systematic characteristics that could be modelled to reduce this variance, though some variance will always be expected in the random effects model due to individual idiosyncrasies.

In contrast to these covariate relationships with the intercept factor variable, the covariate relationships with both pre-recession and recession slopes were almost exclusively non-significant. This suggests that the mean slope in each period was largely descriptive of the mean slope in each covariate category. This was expected for the pre-recession period, but for the recession period this was surprising as some groups have reportedly been more affected by the crisis than others. It may be expected, for example, that young people who have reportedly been hard hit by the economic crisis would experience a greater decline on average than other age groups. The results also suggests that the aggregate decrease in well-being is not only driven by the smaller number who experience negative events such as unemployment for example, but is also felt by the majority who remain employed throughout the study period. Another way of looking at these results is that the time-constant covariates did not sufficiently describe groups of people who had quantitatively different experiences of the recession.

Despite the covariates not being significant, there is strong evidence that people’s *positive psychological health* trajectories varied substantially from the mean over the study period. The model estimated individual trajectories (from the individual-specific variance parameters) indicate that only 23 % of the sample was estimated to have improving *positive psychological health* over the recession period compared to 44 % in the pre-recession period, and only around 1 % showing no change in each period (the distribution of individual-specific variance parameters is presented in Online Resource 3). Focusing on more extreme cases, around 3.6 % of respondents had an estimated trajectory which reduces their *positive psychological health* by over one standard deviation, compared to around 1 % whose estimated trajectories increased by over one standard deviation.

## Discussion

Prior to interpreting the results, a note on effect size is necessary given the abstract scale *positive psychological health* is measured on. *Positive psychological health* during the recession period decreased on average by 0.114 between 2007 and 2010 (i.e. 0.038 per year, S.E. = 0.001). To put the change in context with a comparison frequently found to reduce well-being substantially, the cross-sectional difference between someone who considers themselves disabled compared to someone who does not, was estimated at −0.247 (S.E. = 0.044), just over two times larger than the aggregate decline during the recession period. This is a modest decline of about one fifth of a standard deviation, but for an aggregate effect is by no means substantively insignificant. Behind the aggregate figures, we also found that a reduction in *positive psychological health* became the norm during recession: approximately three-quarters of the sample experienced a negative trajectory in the recession-period, an increase from just over half in the pre-recession period.

The analysis is grounded in a multidimensional conception of well-being which facilitates a more nuanced evaluation in which different indicators represent constituent parts of well-being. By comparing a commonly used measure of SWB with *positive psychological health* using an identical sample and model, our analysis has shown that relying on SWB measures to act as summaries of well-being may be misleading, masking real negative change in the psychological health of the UK working age population. The results corroborated previous research that showed that people’s subjective overall assessments of their well-being remained relatively stable, on average, throughout the economic crisis (Crabtree 2010; Self et al. 2012; ONS 2013). This provides support for the argument that such measures, while useful in many respects, should not be solely relied upon to describe changes in well-being in response to events such as recession. In contrast, the *positive psychological health* measure is a subjective assessment based on six questions which are constant between people and over time, and was therefore put forward as being *less* prone to adaptation. Adaptation was not tested for directly in this study, though provides one explanation of why *life satisfaction* remained stable, while *positive psychological health* decreased. Another potential reason *life satisfaction* measures appear stable could be the annual panel data which may miss shorter-term fluctuations, unlike the daily Gallup Healthways Well-being Index available for the United States. Using *positive psychological health* as another single measure does not overcome the problem of simplifying well-being, but the paper has provided an important empirical example of why single measures are problematic.

The descriptive analysis suggested a steady decrease in *positive psychological health* during the recession period in contrast to a step-change, and the model confirmed that two linear trajectories were a good fit to the data. This pattern contrasts with Deaton’s (2012) findings in United States, where several evaluative and affective subjective measures dropped in late 2008 going into 2009 but had mostly recovered by 2010. This may suggest that unlike the hedonic adaptation noted by Deaton (2012: 15), *positive psychological health* is responding more strongly to peoples lived conditions and circumstances rather than a cognitive response to crisis related events/news. It may also reflect improving underlying economic conditions that saw unemployment in the United States decline throughout 2010. People in employment experienced a decrease in average *positive psychological health*, demonstrating that it was not only the extremely negative experiences of the minority that led to the aggregate worsening. With the unemployment rates continuing to rise throughout 2009 into early 2010, job insecurity and underemployment are potential explanations of worsening *positive psychological health* during the recession period for those who remained in work.

Modelling individual trajectories revealed significant variance in trajectories during the recession. However, there was a lack of significant group-based associations with the estimated trajectories. This may indicate that the mechanisms through which the economic crisis affected people were not clearly differentiated along standard socio-demographic lines. It may also indicate that the covariate operationalisation was suboptimal, specifically the constraint of time-invariant variables, and that discrete events such as becoming unemployed or having a house repossessed are driving more extreme patterns of change. The covariate ‘effects’ are to some extent hiding background structural and contextual factors. Further research into the role of labour market statuses and household structures in moderating the effects of the economic crisis on well-being can to some extent overcome this.

The paper has highlighted that what we measure matters. Using single measures that are often taken as summaries of well-being masks the complexity of the term well-being. In the social policy arena, the appeal of single summary measures can be seen as problematic when the stakes are high. By describing ‘well-being’ as stable during recession there is a risk that the human impact of recession is overlooked.

### Limitations

Limitations of the study arise from the focus on a cohort of eligible respondents at the first wave of the study period who were then followed over six waves. This limits inference in terms of being representative. For example, 16 year olds (as the youngest age eligible) were only represented in the first wave, as these respondents were 17 by wave two and so on. Period effects were assigned to the impact of the recession as per the research objectives, though the design does not rule out unmeasured coinciding period effects. The use of the General Health Questionnaire can be criticised because it contains temporal elements (for example, the answers range from ‘better than usual’ to ‘much less than usual’), which have the potential to make the interpretation of the response unclear. However, these questions are considered sufficient to screen for mental illness and are widely reported as being valid and reliable in panel studies (Goldberg et al. 1997; Pevalin 2000). The use of a derived factor variable to measure the construct of *positive psychological health* from the GHQ questions is also considered an advantage because it aims to measure the underlying construct and is less prone to measurement error.

## Electronic supplementary material

ESM 1 (PDF 207 kb)

## Footnotes

1 The 6 questions are: ‘Here are some questions regarding the way you have been feeling over the last few weeks. For each question please tick the box next to the answer that best describes the way you have felt. Have you recently....1) been able to concentrate on whatever you are doing? 2) felt that you were playing a useful part in things? 3) felt capable of making decisions about things? 4) been able to enjoy normal day-to-day activities? 5) been able to face up to problems? 6) been feeling reasonably happy, all things considered? Response options: i) Better/more so than usual. ii) Same as usual. Iii) Less than usual. iv) Much less than usual.
