# Supplementary material for: Well-Being During Recession in the UK
Source: Appl Res Qual Life. 2016 Apr 29;12(2):369–87. doi: 10.1007/s11482-016-9465-8 (PMC5435781; doi:10.1007/s11482-016-9465-8)

Online Resources (1, 2 & 3) for 'Well-being During Recession in the UK', *Applied Research in Quality of Life*

Online Resource 1 Sample characteristics.

Time-constant variable	Wave 1 (start of pre-recession period)	Wave 4[e] (start of recession period)
	proportion	*proportion*
***Sex**:* male	.49	.49
Female	.51	.51
***Age band** [a]:* 16-24	.08	.09
25-34	.19	.20
35-49	.40	.43
50-59/64	.33	.28
***Labour market status** [c]:* Employed	.66	.65
Unemployed	.003	.002
Economically inactive	.05	.04
In and out of employment	.26	.27
Between unemployed and inactive	.03	.03
***Children in household** [b]:* No children	.63	.59
Child(ren)	.37	.41
***Marital status** [b]:* Couple	.71	.71
Single	.20	.20
Ex-couple	.08	.08
Widow/widower	.01	.01
***Educational attainment** [b]:* High	.20	.21
Intermediate	.69	.69
No qualifications	.11	.10
***Tenure** [b]:* Owned: outright	.21	.18
Owned: mortgage	.58	.61
Rent: social	.12	.12
Rent: private/other	.09	.09
***Disability** [b]:* Not disabled	.95	.95
Considers self disabled	.05	.05
***Household income (monthly)** [d]*	*mean*	*mean*
Actual household income	£3,605	£3,696
Logged household income	8.05	8.09
Unlogged equivalent of logged hh income	£3,124	£3,252

Population aged 16-59/64 (F/M). BHPS UK sample, wave 2004 and 2007.
[a]Status in 2007. [b]Status in 2007, or the closest preceding, then subsequent non-missing wave. [c]Groups 'Employed', 'Unemployed' and 'Economically inactive' refer to cases where the nominal labour market status was constant across all waves (allowing for missing observations). 'In and out of employment' describes cases who changed from employment to non-employed status, or vice versa, at least once. 'Between unemployed and inactive' describes cases who changed from unemployment to inactivity, or vice versa, at least once, and who were never employed in the study period. [d]Household level income averaged across study period (uses 'real' income).
[e]Note that a change in proportion across waves is acheived only by missing data and attrition as the variables are time-constant.

Online Resource 2 Covariate Coefficients for Model 1, Piecewise Latent Curve Model for *Life Satisfaction.*

	Intercept		Pre-recession slope		Recession slope	
	Estimate	(S.E.)	Estimate	(S.E.)	Estimate	(S.E.)
**Sex**: Men [a]						
Women	0.040	(0.027)	-0.004	(0.010)	-0.001	(0.016)
**Age band**: 16-24 [a]						
25-34	-0.225*	(0.065)	0.063*	(0.026)	-0.054	(0.035)
35-49	-0.400**	(0.071)	0.041	(0.028)	-0.040	(0.038)
50-59/64	-0.329**	(0.072)	0.065*	(0.027)	-0.060	(0.042)
**Labour market and employment status**: Employed[a]						
Unemployed	-0.254	(0.292)	-0.033	(0.093)	0.036	(0.230)
Economically inactive	-0.501**	(0.100)	0.017	(0.030)	-0.024	(0.052)
In and out of employment	-0.200**	(0.037)	0.022	(0.013)	-0.031	(0.019)
Between unemployed and inactive	-0.350*	(0.116)	-0.055	(0.040)	-0.092	(0.054)
**Children in household**: No children [a]						
Children	0.030	(0.038)	-0.021	(0.013)	-0.032	(0.017)
**Marital status**: Couple [a]						
Single	-0.310**	(0.052)	0.018	(0.019)	0.006	(0.030)
Ex-couple	-0.543**	(0.062)	0.065*	(0.025)	0.030	(0.036)
Widow/widower	-0.500**	(0.124)	-0.062	(0.060)	0.100	(0.132)
**Educational attainment**: High [a]						
Intermediate	-0.001	(0.035)	0.000	(0.013)	-0.029	(0.018)
No qualifications	0.057	(0.065)	-0.034	(0.026)	0.019	(0.034)
**Tenure**: Owned: outright [a]						
Owned: mortgage	-0.078*	(0.039)	-0.013	(0.015)	0.010	(0.024)
Rent: social	-0.245**	(0.067)	-0.020	(0.026)	0.021	(0.034)
Rent: private/other	-0.268**	(0.064)	0.020	(0.024)	-0.021	(0.036)
**Disability**: Not disabled [a]						
Considers self disabled	0.381	(0.094)	-0.050	(0.033)	0.026	(0.040)
**Household income (centred) (log)**	0.093*	(0.031)	0.015	(0.012)	0.043*	(0.019)

[a]= reference category

**p<0.05 **p<0.0005. N=10,254. Population aged 16-59/64 (F/M). BHPS UK sample, wave 2004 to waves 2008/9 and Understanding Society BHPS cohort wave 2010. Weighted estimates.*

Online Resource 3 Distribution of predicted slope coefficients (showing rate of change per year) for slope 1 (pre-recession period, top panel) and slope 2 (recession period, bottom panel), from Model 2 for *positive psychological health*

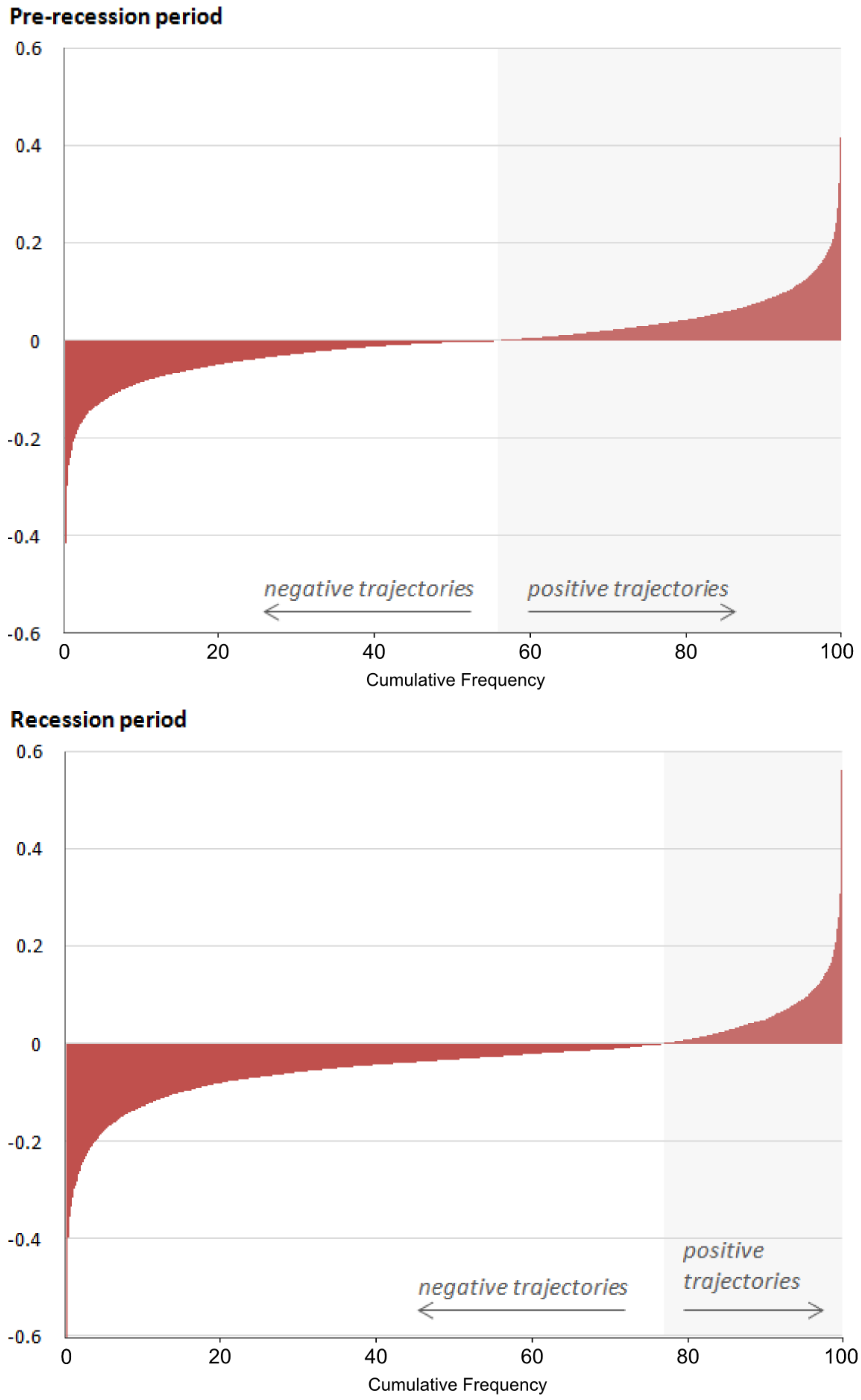

Supplement: Supplementary file 1 — (PDF 207 kb) [file 11482_2016_9465_MOESM1_ESM.pdf]
